# Obesity/Type II Diabetes Promotes Function-limiting Changes in Murine Tendons that are not reversed by Restoring Normal Metabolic Function

**DOI:** 10.1038/s41598-018-27634-4

**Published:** 2018-06-15

**Authors:** Valentina Studentsova, Keshia M. Mora, Melissa F. Glasner, Mark R. Buckley, Alayna E. Loiselle

**Affiliations:** 1 Center for Musculoskeletal Research, University of Rochester Medical Center, Rochester, NY USA; 2 Department of Biomedical Engineering, University of Rochester, Rochester, NY USA; 3 Cell Biology of Disease Graduate Program, University of Rochester Medical Center, Rochester, NY USA; 4 Department of Orthopaedics & Rehabilitation, University of Rochester Medical Center, Rochester, NY USA

## Abstract

Type II Diabetes (T2DM) negatively alters baseline tendon function, including decreased range of motion and mechanical properties; however, the biological mechanisms that promote diabetic tendinopathy are unknown. To facilitate identification of therapeutic targets we developed a novel murine model of diabetic tendinopathy. Mice fed a High Fat Diet (HFD) developed diet induced obesity and T2DM and demonstrated progressive impairments in tendon gliding function and mechanical properties, relative to mice fed a Low Fat Diet (LFD). We then determined if restoration of normal metabolic function, by switching mice from HFD to LFD, was sufficient to halt the pathological changes in tendon due to obesity/T2DM. However, switching from a HFD to LFD resulted in greater impairments in tendon gliding function than mice maintained on a HFD. Mechanistically, IRβ signaling is decreased in obese/T2DM murine tendons, suggesting altered IRβ signaling as a driver of diabetic tendinopathy. However, knock-down of IRβ expression in S100a4-lineage cells (IRcKO^S100a4^) was not sufficient to induce diabetic tendinopathy as no impairments in tendon gliding function or mechanical properties were observed in IRcKO^S100a4^, relative to WT. Collectively, these data define a murine model of diabetic tendinopathy, and demonstrate that restoring normal metabolism does not slow the progression of diabetic tendinopathy.

## Introduction

Tendons are dense connective tissues, composed primarily of type I collagen, that transmit forces between muscle and bone and are required for skeletal locomotion.

Type II Diabetes Mellitus (T2DM) is a metabolic disease characterized by hyperglycemia and a decrease in sensitivity to insulin^1^, and is strongly associated with obesity^2^. While it is difficult to dissect the systemic effects of Type II Diabetes from obesity, it is clear that induction of both obesity/T2DM leads to severe alterations in metabolic function resulting in a variety of pathological changes throughout the body^3^.

While T2DM results in a plethora of systemic pathologies^4^, the dramatic impact of T2DM on the musculoskeletal system has only more recently become appreciated. Approximately 83% of patients with T2DM will experience some form of musculoskeletal system degeneration or inflammation^5^. Furthermore, T2DM has been shown to increase the risk of tendinopathy and tendonitis^6^, with a variation in sensitivity to T2DM between different tendons^6,7^. The flexor tendons (FTs) of the hand are among the most sensitive to pathological changes of T2DM, with approximately 50% of T2DM patients experiencing limited hand function and mobility^8,9^. FTs facilitate movement of the hand and digits via nearly frictionless gliding of the FTs through the synovial sheath. Diabetic tendinopathy results in severe inflammation and pain in the hand and digits^10^, and decreased digit range of motion (ROM)^6^. These impairments in tendon function can severely impair use of the hands, and subsequently diminish overall quality of life. At present, the only treatments for diabetic tendinopathy are surgical release of fibrous adhesions, or corticosteroid injections to reduce local inflammation^11,12^. Consistent with disruptions in tendon homeostasis, diabetic tendons are more likely to experience spontaneous rupture requiring surgical repair^11,12^. Furthermore, tendon healing after acute injury is impaired in diabetic patients relative to non-diabetic patients^13^. Therefore, there is a strong need to understand the underlying pathology of diabetic tendinopathy in order to develop therapeutic interventions to prevent disease progression.

Currently, very little is known about the cellular and molecular mechanisms underlying diabetic tendinopathy, although pathological changes increase as a function of T2DM disease duration^6^. To begin to address this gap in knowledge, we have utilized a murine model of diet induced obesity and Type II diabetes^14^ to test the hypothesis that flexor tendons from obese/diabetic mice will undergo progressive, pathological changes in tendon gliding function and mechanical properties. We also examined the effects of restoration normal metabolic function as a potential means to reverse or halt diabetic tendinopathy. Finally, given the loss of insulin sensitivity that was observed in obese/T2DM tendons, we generated tendon-specific Insulin Receptor (IR) conditional knockout mice to determine if IR deletion in the tendon is sufficient to recapitulate the diabetic tendinopathy phenotype.

## Results

### HFD induces Obesity and Type II Diabetes in Mice

**Figure 1:**
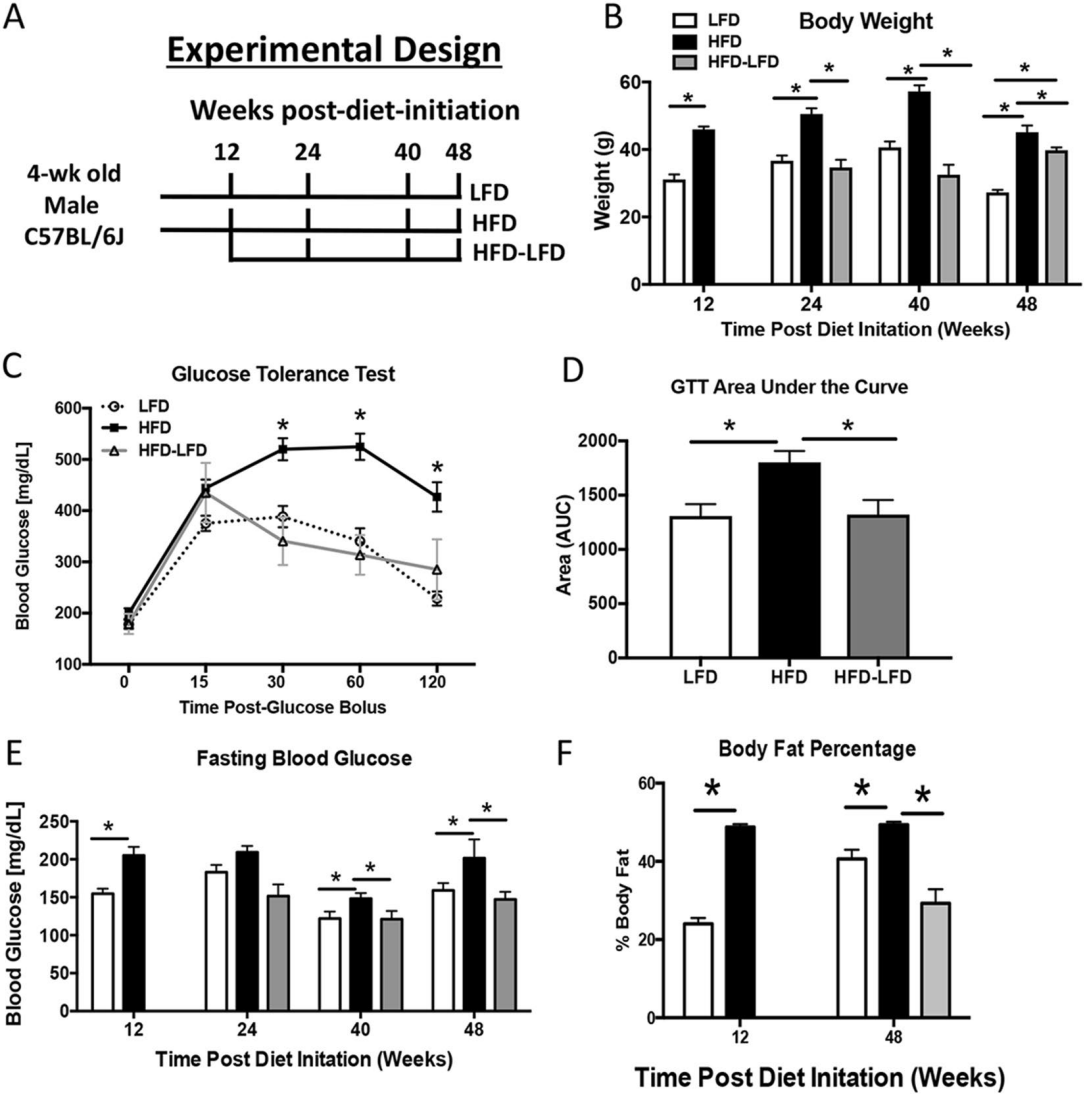
A High Fat Diet induces obesity and Type II Diabetes Mellitus. (**A**) Male C57Bl/6 J mice were initiated on either a High Fat Diet (HFD) or Low Fat Diet (LFD) at 4 weeks of age. 12 weeks after diet initiation half of the HFD cohort was randomly selected and switched to a LFD. Animals were harvested at 12, 24, 40, and 48 weeks after diet initiation (12–36 weeks after switching to LFD for the HFD-LFD cohort. (**B**) Body weight was measured in LFD, HFD mice between 12 and 48 weeks after diet initiation. HFD-LFD mice were initiated on a HFD, and switched to a LFD 12 weeks after initiation, as such body weights from these mice were measured only between 24–48 weeks (12–36 weeks after switching to the LFD). (**C**) At 20 weeks after diet initiation a significant impairment in glucose tolerance was observed in HFD, compared to LFD and HFD-LFD. A sustained increase in glucose levels post-glucose bolus is indicative of impaired glucose tolerance. (**D**) Quantification of Area under the Curve (AUC) from Glucose Tolerance Test (GTT). (**E**) Changes in fasting blood glucose were measured after a 5 hr fast between 12–48 weeks after diet initiation. (**F**) Body fat percentages of LFD, HFD, and HFD-LFD mice at 12 and 48 weeks post diet initiation. *Indicates p < 0.05.

HFD mice had a significant increase in body weight relative to LFD and HFD-LFD at all time-points, (24 weeks- LFD: 33.75 g ± 2.27, HFD: 49.05 g ± 2.24; HFD-LFD: 28.8 g ± 7.03, p < 0.0001) (Fig. 1B). Body weights of HFD-LFD mice were not significantly different than LFD between 24–40 weeks, however by 48 weeks a significant increase in body weight was observed in HFD-LFD mice, relative to LFD. Glucose tolerance was significantly impaired in HFD, relative to LFD during a glucose tolerance test, with a 37% increase in area under the curve (AUC) in HFD, relative to LFD at 20 weeks post diet initiation (p < 0.0001) (Fig. 1C). In contrast, no change in glucose tolerance was observed between HFD-LFD and LFD at this time (Fig. 1C). Fasting BG was significantly increased in HFD mice at 12, 40 and 48 weeks, relative to LFD (12 weeks- LFD: 154.6 mg/dL ± 16.1, HFD: 205.2 mg/dL ± 24.7, p < 0.0001). However, this increase was not observed in HFD-LFD compared to LFD between 24–48 weeks (Fig. 1D). Significant increases in body fat percentage were observed in HFD, relative to LFD at 12 weeks and 48 weeks (12 weeks: +100%, p < 0.0001; 48 weeks: +21%, p = 0.004). Body fat percentage was significantly decreased in HFD-LFD mice, relative to both HFD (−68%, p = 0.0002), and LFD (−27%, p = 0.03) at 48 weeks (Fig. 1E).

### HFD and HFD-LFD Impairs Tendon Range of Motion

**Figure 2:**
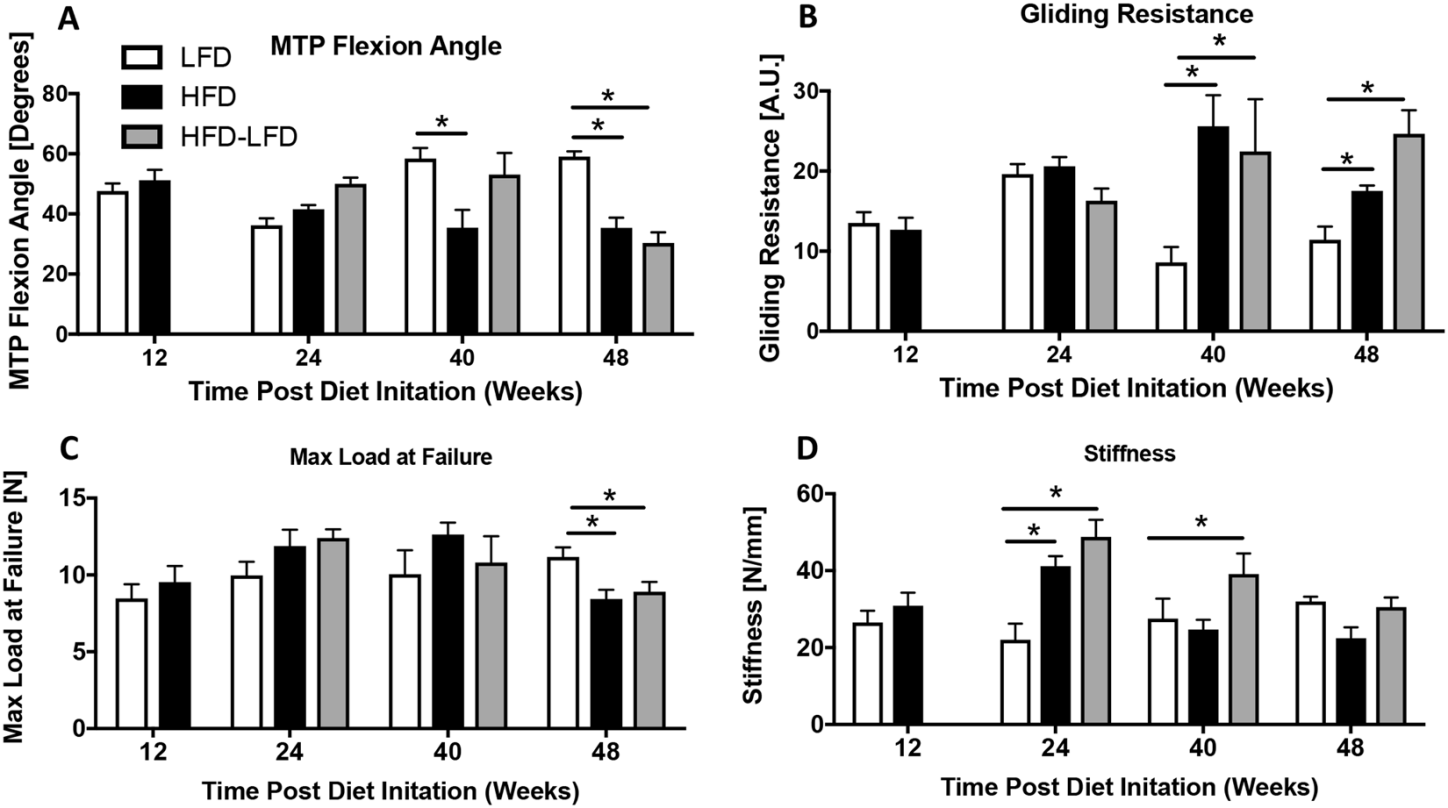
T2DM/obesity alters tendon gliding function and mechanical properties. (**A**) Measurement of metatarsophalangeal (MTP) joint flexion angle, (**B**) Gliding Resistance, (**C**) Maximum load at failure, and (**D**) Stiffness of the FDL tendon in LFD, HFD and HFD-LFD tendons between12–48 weeks after diet initiation. *Indicates p < 0.05.

To assess tendon range of motion we quantified metatarsophalangeal (MTP) flexion angle and gliding resistance. No change in MTP Flexion Angle was observed between groups at 12 and 24 weeks. However, at 40 and 48 weeks the MTP Flexion Angle was significantly decreased in HFD mice relative to LFD (48 weeks- LFD: 59.07° ± 5.0; HFD: 35.4° ± 9.52, p < 0.0001). At 48 weeks, the MTP Flexion angle was significantly decreased in HFD-LFD relative to LFD (HFD-LFD: 30.36° ± 9.3, p = 0.04) (Fig. 2A). Similar to MTP flexion angle between groups at earlier time-points, HFD and HFD-LFD at 12 and 24 weeks showed no significant changes in gliding resistance. However at 40, and 48 weeks, gliding resistance was significantly increased in HFD and HFD-LFD, relative to LFD (48 weeks- LFD: 11.4 ± 3.72; HFD: 17.48 ± 1.37; HFD-LFD: 24.64 ± 7.83, p < 0.05) (Fig. 2B).

### HFD and HFD-LFD alters tendon mechanical properties

Maximum load at failure was not significantly different between groups at 12, 24 and 40 weeks. At 48 weeks max load at failure was significantly decreased in HFD and HFD-LFD tendons, relative to LFD (HFD: −25%, p < 0.01; HFD-LFD: −21%, p < 0.04) (Fig. 2C). No change in tendon stiffness was observed between groups at 12 weeks. At 24 weeks both HFD and HFD-LFD tendons exhibited a significant increase in stiffness relative to LFD (+49% and +117%, respectively, p < 0.006). At 40 weeks a significant increase in stiffness was observed in HFD-LFD tendons, relative to LFD, while no differences in stiffness were observed between HFD and LFD at this time, or between any groups at 48 weeks post-diet initiation (Fig. 2D).

### Obesity/ T2DM Alters Collagen Fibril Size Distribution

**Figure 3:**
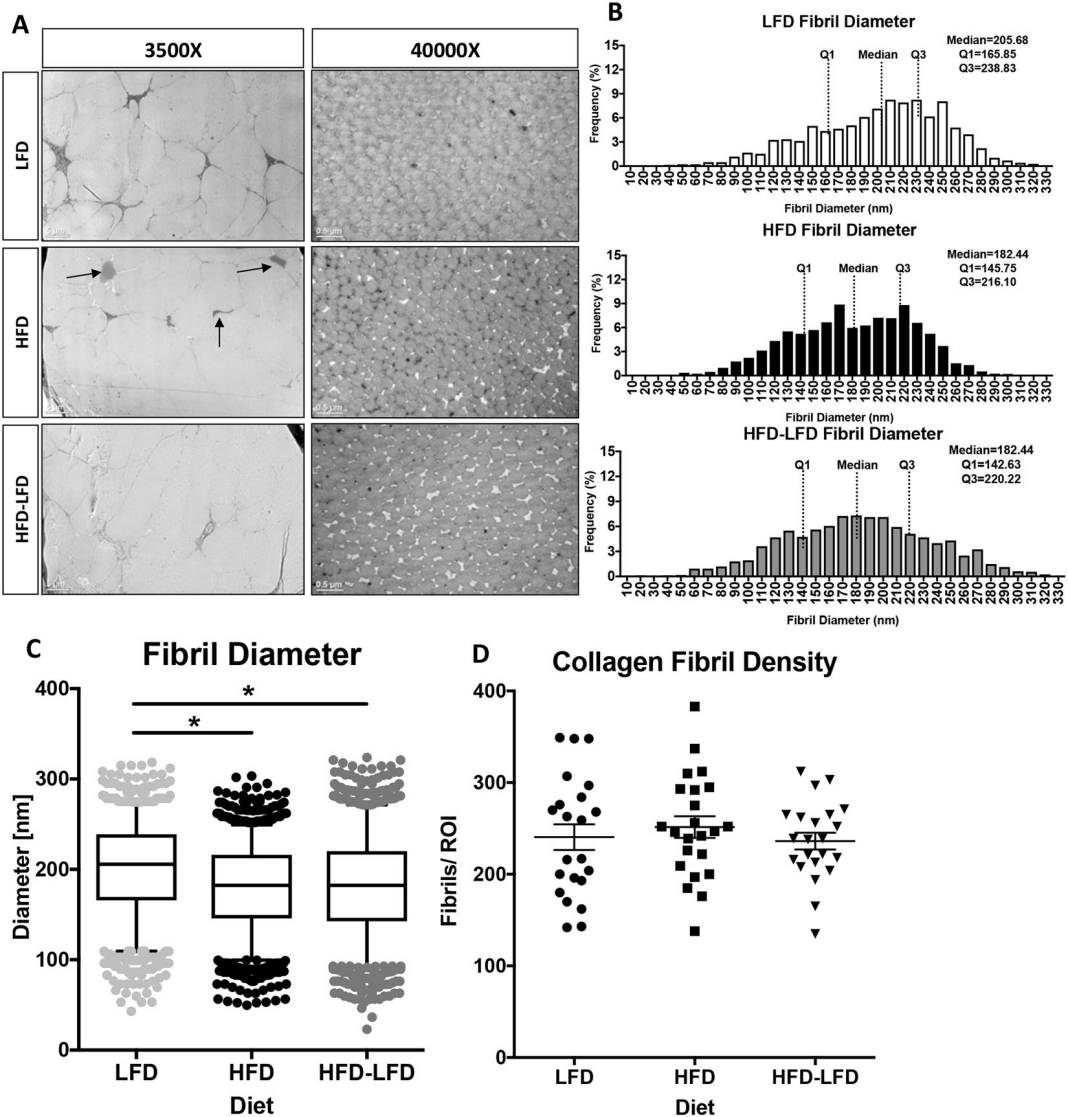
T2DM/obesity alters collagen fibril diameter. (**A**) TEM axial images of the FDL tendon from LFD, HFD and HFD-LFD mice at 40 weeks post diet initiation. Lipid deposits in HFD tendons are noted by black arrows. Scale bars represent 5 microns in 3500x magnification images, and 0.5microns in the 40,000x magnification images. (**B**) Collagen fibril diameter histograms demonstrating a decrease in median fibril diameter in HFD and HFD-LFD tendons compared to LFD. (**C**) Collagen fibril diameter distributions with boxplot whiskers spanning data between the 5^th^ and 95^th^ percentiles. Data outside this range are plotted as individual points. (**D**) Collagen fibril density. *Indicates p < 0.05.

To define the impact of T2DM on collagen fibril density and compactness we utilized Transmission Electron Microscopy (TEM) at 40 weeks post-diet initiation. At 3500x magnification, there were no observed changes in collagen packing and organization between LFD, HFD and HFD-LFD. However, lipid deposits were observed in the mid-substance of HFD tendons (black arrows, Fig. 3A). At 40,000x magnification there was a significant alteration in median collagen fibril diameter in HFD and HFD-LFD, relative to LFD (Fig. 3A–C). The median fibril diameter decreased from 200.1 nm in LFD to 180.4 nm in HFD (p = 0.028), and 182.1 nm in HFD-LFD (p = 0.046) (Fig. 3B,C). No change in collagen fibril density was observed between groups at 40 weeks post-diet initiation (Fig. 3D).

### Insulin Receptor Signaling is attenuated in HFD tendons

**Figure 4:**
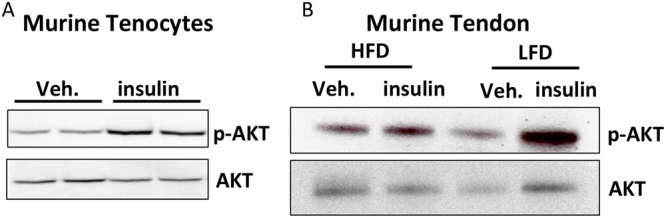
Insulin receptor expression and signaling are altered in diabetic tendons. (**A**) Primary tenocytes isolated from non-diabetic murine tendons demonstrate strong expression of p-Akt relative to vehicle treated tenocytes, indicating activation of IR signaling in tenocytes. (**B**) Tendons from HFD and LFD mice were stimulated with insulin or vehicle (0.5% BSA in PBS). Activation of IR signaling was observed in LFD tendons based on the increased expression of p-Akt. In contrast, no increase in p-Akt expression was observed in insulin stimulated HFD tendons compared to vehicle treatment indicating blunted sensitivity to insulin in HFD tendons. Blots were probed for p-Akt and imaged, following stripping of the membrane the same blots were then probed for total Akt. Full length blots are presented in Supplementary Figure 1.

Based on changes in insulin sensitivity in other diabetic tissues^15^, we examined changes in activation of Insulin Receptor (IR) signaling in primary tenocytes and obese/T2DM and non-diabetic murine tendons. Primary tenocytes demonstrate a robust activation of IR signaling, as indicated by increased p-Akt expression, relative to vehicle treated tenocytes (Fig. 4A). Upon insulin stimulation tendons from LFD mice demonstrated a robust induction of p-Akt expression, indicating activation of IR signaling. In contrast, induction of p-Akt was blunted in HFD tendons (Fig. 4B).

### S100a4-Cre results in decreased insulin receptor expression in the tendon

**Figure 5:**
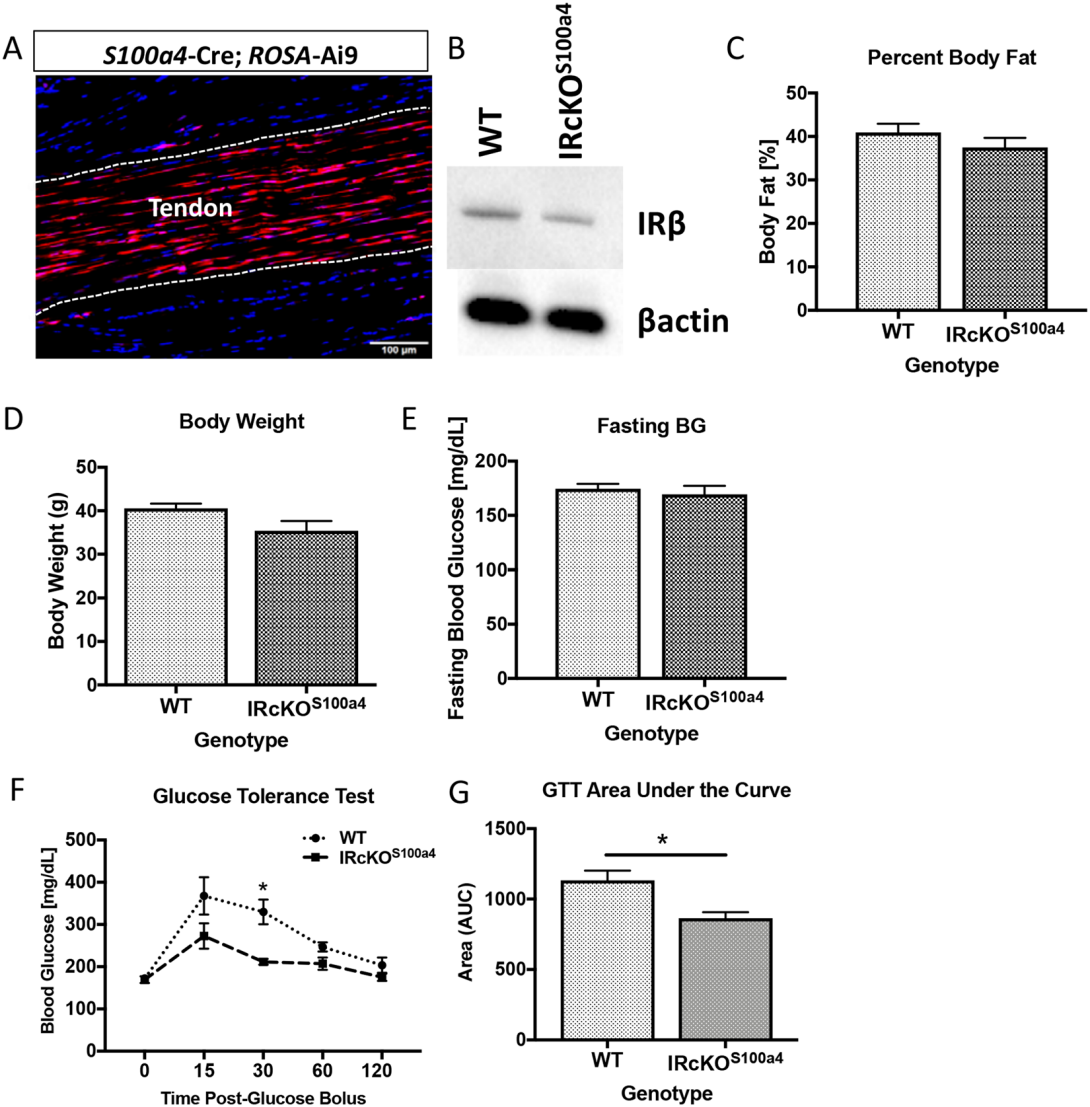
IRβ deletion in S100a4-lineage cells does not induce obesity or T2DM. (**A**) The recombination efficiency of S100a4-Cre in the tendon was visualized using the Ai9 reporter and demonstrates efficient recombination in the tendon. (**B**) IRcKO^S100a4^ decreases IRβ protein expression in tendon, relative to WT. Full length blots are presented in Supplementary Figure 2. (**C**–**E**) No changes in (**C**) body weight, (**D**) percent body fat, or (**E**) fasting blood glucose were observed between WT and IRcKO^S100a4^ mice at 48 weeks of age. (**F**–**G**) IRcKOS100a4 had more efficient restoration of blood levels during the glucose tolerance test (**F**), and as calculated by quantification of Area Under the Curve from the glucose tolerance test (**G**).

Given the loss of insulin sensitivity in obese/T2DM murine tendons and the increase in IRβ expression in human T2DM tendons, we examined the effects of IRβ deletion in the tendon using S100a4-Cre. To demonstrate that S100a4-Cre efficiently targets resident tenocytes, S100a4-Cre^+^; Ai9 reporter mice were used. Expression of Ai9 fluorescence was observed in nearly all resident tenocytes (Fig. 5A). Furthermore, western blot analyses demonstrated a decrease in IRβ expression in S100a4-Cre^+^; IR^F/F^ mice (IRcKO^S100a4^), relative to wild type (WT) littermates (Fig. 5B). No changes in body fat percentage (Fig. 5C), body weight (Fig. 5D), or fasting blood glucose (Fig. 5E) were observed between WT and IRcKO^S100a4^ mice. A transient increase in glucose tolerance was observed in IRcKO^S100a4^ mice at 30 minutes after administration of the glucose bolus, however no difference in glucose tolerance was observed at 15, 60 or 120 minutes (Fig. 5F). A significant, 23% decrease in Area under the Curve was observed in IRcKO^S100a4^ mice during the glucose tolerance test (Fig. 5G). Thus, IRcKO^S100a4^ allows examination of the specific effects of IRβ knock-down in the tendon independent of obesity/T2DM.

### Insulin receptor deletion in S100a4-lineage cells does not induce diabetic tendinopathy

**Figure 6:**
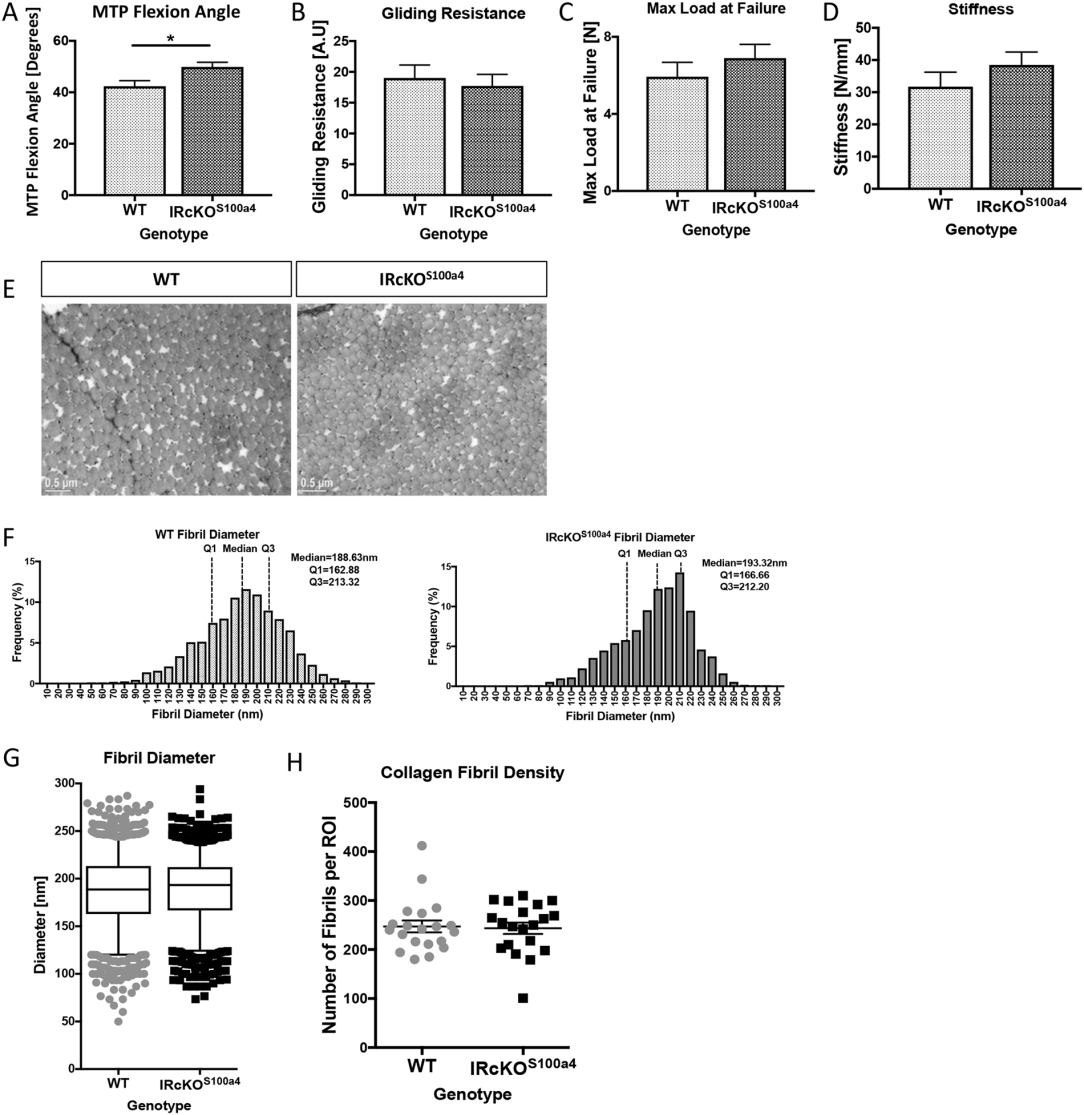
Deletion of IRβ in S100a4-lineage cells does not impair tendon gliding function, mechanical properties or collagen organization. (**A**) MTP ROM, (**B**) Gliding Resistance, (**C**) Max load at failure and (**D**) Stiffness were assessed at 48 weeks of age in WT and IRcKO^S100a4^ tendons. *Indicates p < 0.05. (**E**) TEM axial images of the FDL tendon from WT and IRcKO^S100a4^ at 48 weeks of age. (**F**) No changes in collagen fibril diameter were observed between WT and IRcKO^S100a4^ tendons. (**G**) Collagen fibril diameter distributions with boxplot whiskers spanning data between the 5^th^ and 95^th^ percentiles. Data outside this range are plotted as individual points. (**H**) Collagen fibril density of TEM images of tendons from WT and IRcKO^S100a4^ mice at 48 weeks of age.

Tendons from IRcKO^S100a4^ had a significant increase in MTP Flexion Angle (49.87° ± 1.79, p = 0.02) at 48 weeks, relative to WT (42.37° ± 2.16) (Fig. 6A). No change in gliding resistance was observed between WT and IRcKO^S100a4^ (WT: 19.02 ± 2.1; IRcKO^S100a4^: 17.73 ± 1.87, p > 0.05) (Fig. 6B). Maximum load at failure and stiffness were not significantly different between IRcKO^S100a4^ and WT tendons (Max load: WT: 5.92 N ± 0.74, IRcKO^S100a4^: 6.9 N ± 0.7, p = 0.36) (Fig. 6C,D). Furthermore, TEM analyses demonstrate that loss of IRβ in S100a4-lineage cells did not alter collagen fibril diameter distribution (WT Median: 188.63; IRcKO^S100a4^ Median: 193.32, p = 0.21) (Fig. 6E–G), or fibril density (Fig. 6H) (p = 0.82) at 48 weeks of age.

## Discussion

Both Type I and Type II Diabetes patients are at an increased risk of developing tendinopathy^9^. While several tendons can be affected by diabetic tendinopathy, including the Achilles and the supraspinatus tendons, the flexor tendons are most commonly impacted by T2DM^8,9^. Diabetic tendinopathy decreases tendon strength and increases the likelihood of tendon rupture^11^. Currently, treatment options for diabetic tendinopathy are limited to corticosteroid injections, or surgical intervention^11,12^. Therefore it is imperative to understand the biological mechanism driving this pathology in order to develop novel therapeutic approaches to improve tendon function. To address the gap in knowledge surrounding the progression of diabetic tendinopathy, we defined the effects of diet induced obesity and Type II diabetes on tendon function. We have demonstrated that induction of T2DM/obesity recapitulates the phenotypes observed clinically in diabetic flexor tendinopathy, including decreased mechanical properties and tendon range of motion^6,11,12^. Furthermore, profound changes in collagen organization in both HFD and HFD-LFD tendons support a strong link between altered ECM organization and impaired mechanical properties in diabetic tendinopathy. Perhaps most interestingly, we demonstrate that resolution of metabolic dysfunction from obesity/T2DM is insufficient to reverse diabetic tendinopathy, highlighting the potential need to intervene directly at the tendon cell/tissue level, rather than simply managing or reversing T2DM.

Clinically, one of the pathological hallmarks of diabetic tendinopathy is a decrease in tendon strength, rendering the tendon more susceptible to rupture^11,12^, while others have shown that diabetic tendons are stiffer than non-diabetic^16^. Several studies have demonstrated that hyperglycemia is associated with inferior mechanical properties. Maffulli *et al*., demonstrated that mechanical properties are reduced in the Achilles and supraspinatus tendons in a hyperglycemic environment^17^, while impairments in restoration of tendon mechanical properties after injury is delayed in the Achilles tendon of hyperglycemic rats^18^. Consistent with this, we observed a significant decrease in tensile strength of the FT at 48 weeks in both the HFD and HFD-LFD tendons relative to LFD, although it is not yet clear to what extent these phenotypes are driven by elevated blood glucose levels, relative to other components of metabolic dysfunction that occur in obese/diabetic mice. Interestingly, we observed transient, significant increases in HFD (24 weeks) and HFD-LFD (24 and 40 weeks) tendons, relative LFD, consistent with clinical reports^16^. However, the effects of T2DM on tendon stiffness in pre-clinical models are inconsistent. No changes were observed between HFD and LFD at 40 weeks due to a dramatic decrease in stiffness of HFD tendons between 24–40 weeks. Stiffness progressively decreased in HFD-LFD tendons with no differences observed between groups at 48 weeks. Interestingly, Connizzo *et al*., have demonstrated a decrease in tendon stiffness in diabetic animals at the insertion, however no changes were observed in the tendon midsubstance^7^, while Boivin *et al*., observed a significant decrease in stiffness of tendons from db/db diabetic mice, relative to non-diabetic controls^19^. These differential effects may be explained in part by differences in duration of T2DM, and tendon-specific differences in responsive to T2DM. Furthermore, a greater understanding of how T2DM alters the collagen ECM during the progression of diabetic tendinopathy may help explain both the changes in stiffness over time observed in this model, and the different phenotypes observed in other studies.

Tendon mechanical properties and ROM are dependent on collagen ECM organization^20^. Given the decrements in mechanical properties of tendons from HFD and HFD-LFD mice, we examined changes in collagen organization and fibril diameter. TEM demonstrated significant changes in collagen fibril diameter distribution in HFD and HFD-LFD tendons, relative to LFD. These observed changes in fibril size may alter tendon gliding function and suggests altered collagen fibril architecture as a mechanism of impaired tendon function in diabetic tendinopathy. Consistent with this, an increase in tendon bulk is observed clinically in type II diabetic patients^21^, suggesting that loss of collagen compactness may impede normal gliding function leading to decreased tendon functionality and range of motion.

At the molecular level, the mechanisms driving diabetic tendinopathy are unclear. However, several studies have suggested that Advanced Glycation Endproducts (AGEs), which are dramatically increased in T2DM tissue^22^, may promote loss of collagen organization due to altered cross-linking resulting from AGE-collagen binding^23^. However, Fessel *et al*., have recently demonstrated that AGEs are unable to induce tissue level impairments in tendon mechanical properties^24^, suggesting other mechanisms may be driving this phenotype. To that end we have demonstrated that tendon is an insulin target tissue, and that obesity/T2DM results in a substantial decrease in insulin sensitivity in HFD tendons at 48 weeks, suggesting that loss of IR signaling in HFD tendons may promote diabetic tendinopathy. Contrary to our hypothesis, conditional deletion of IRβ in S100a4-lineage cells did not promote changes in tendon matrix organization, gliding function or mechanical properties. However, deletion of IRβ in S100a4-lineage cells results in only a knock-down of IRβ expression, thus, we do not know what the effects of complete IRβ deletion are, or how IRβ deletion in different cell types may alter tendon structure and function. The low degree of IRβ knock-down is particularly interesting given the high efficiency we have observed in with the S100a4-Cre in other models^25^ and suggests that a population other than S100a4-lineage cells are the predominant IRβ expressing cell population. For example, it is not clear what the relationship between S100a4^+^ resident tenocytes and Scx^+^ resident tenocytes are, and how the effects of IR deletion in Scx^+^ cells may differ from IRcKO^S100a4^. Moreover, given the heightened systemic inflammation of obesity/diabetes, there is likely a local increase in inflammatory cells such as macrophages that may also be involved in modulating IR signaling.

While we clearly demonstrate that a murine model of diet induced obesity and type II Diabetes recapitulates many of the clinical findings of diabetic tendinopathy, there are several limitations that must be considered. We have confined these studies to male mice, as male C57Bl/6 J mice are susceptible to diet induced obesity and T2DM, while females become obese but not diabetic^26,27^. While we have shown that knock-down of IRβ in S100a4-lineage cells is insufficient to recapitulate the phenotypes observed in obese/T2DM mice, future studies will be needed to determine if IRβ deletion, in the context of obesity, accelerates or slows tendinopathy development. We have also initiated the development of diet-induced obesity and T2DM in juvenile mice rather than adult mice, which may impact the severity or development of the phenotypes observed in our model. However, given that the incidence of obesity/T2DM is rapidly rising in the pediatric population^28^, modeling this phenomenon is scientifically justified and understanding how age of onset affects diabetic tendinopathy progression is an important focus for future studies. Finally, while we have demonstrated the obesity/T2DM alters tendon structural mechanical properties, we have not determined the effects on material properties. Given the crucial contribution of material properties to function, it will be important to define these potential changes in future studies.

In summary, obesity/T2DM is sufficient to induce an irreversible cascade of pathologic changes in the flexor tendon including altered ECM organization, ultimately leading to diminished mechanical properties and tendon ROM. Furthermore, IRβ deletion in S100a4-lineage cells does not alter tendon ECM organization or tendon function. Given that diminished insulin sensitivity is observed in obese/T2DM tendons, future investigation in the role of IRβ in additional cell populations will be important. Finally, resolution of metabolic dysfunction was not sufficient to prevent disease progression in the tendon, suggesting that understanding the the tendon-specific cell and molecular response to obesity/T2DM will be critical to the successful management and treatment of diabetic tendinopathy.

## Methods

### Animal Ethics

This study was carried out in strict accordance with the recommendations in the Guide for the Care and Use of Laboratory Animals of the National Institutes of Health. All animal procedures were approved by the University Committee on Animal Research (UCAR) at the University of Rochester (UCAR Number: 2014-004).

### Diet Induced Obesity and Type II Diabetes

Male C57Bl/6J mice (Jackson Laboratories, Bar Harbor, ME) were housed in groups of five in a 12-hour light/dark cycle. At four weeks of age mice were placed on either a low fat diet (LFD; 10% Kcal, Research Diets #12450 J), or high fat diet (HFD; 60% Kcal, Research Diets #D12492) for up to 48 weeks. A third cohort (HFD-LFD) of mice was placed on the HFD for 12 weeks, and then switched to the LFD until sacrifice (Fig. 1A). Mice were sacrificed at 12, 24, 40, and 48 weeks post-diet initiation, via carbon dioxide inhalation and cervical dislocation.

### Insulin Receptor conditional deletion mice

To drive loss of IRβ in the tendon, IRβ^flox/flox^ (#6955, Jackson Laboratories)^29^, were crossed to *S100a4*-Cre mice (#12641, Jackson Laboratories), resulting in IRcKO^S100a4^ mice. Cre-; IRβ^flox/flox^ littermates were used as wild type (WT) controls. To confirm efficient targeting of the tendon, *S100a4*-Cre mice were crossed to Ai9 reporter mice (#7909, Jackson Laboratories)^30^, resulting in tdTomato fluorescence upon Cre-mediated recombination. Mice were sacrificed at 48 weeks of age.

### Measurement of Fasting Blood Glucose

At 12, 24, 40 and 48 weeks post-diet-initiation, mice (n = 6 per diet per time-point) were fasted for five hours^31^, weighed, and fasting blood glucose levels were measured using a OneTouch2 blood glucometer (LifeScan Inc. Milpitas, CA). Fasting blood glucose was measured at 48 weeks of age in IRcKO^S100a4^ and WT mice (n = 6 per genotype).

### Glucose Tolerance Test

To assess glucose tolerance at 20 weeks post-diet-initiation LFD, HFD and HFD-LFD mice (n = 6 per diet) received a 10 uL/g bolus of 20% glucose in PBS via intraperitoneal injection following a five hour fast. Blood glucose levels were measured at 15, 30, 60 and 120 minutes after the glucose bolus. Glucose tolerance was assessed at 48 weeks of age in IRcKO^S100a4^ and WT mice.

### Assessment of Body Fat

At 12 and 48 weeks post-diet-initiation (48 weeks of age in IRcKO^S100a4^ and WT mice) the percent body fat was measured using the PIXImus dual-energy X-ray absorptiometer (DXA) (GE Lunar PIXImus, GE Healthcare, WI).

### Assessment of Gliding Function

Following sacrifice, hind limbs were harvested for gliding and biomechanical testing at 12, 24, 40, and 48 weeks post diet initiation (n = 6–10 per diet per time-point.) IRcKO^S100a4^ and WT mice were harvested at 48 weeks of age (n = 8–10). The FDL tendon was isolated from the myotendinous junction to the tarsal tunnel, leaving the tendon intact through the digits. The proximal end of the tendon was secured between two pieces of tape using cyanoacrylate. The tibia was secured using a custom grip and the proximal end of the FDL was incrementally loaded from 0–19 g. Upon application of each weight, an image was taken of the metatarsophalangeal (MTP) joint flexion, and measured using ImageJ (http://imagej.net). Using these images, MTP flexion angle was calculated as the difference in MTP flexion angle between unloaded (0 g) and 19 g. The gliding resistance was calculated by fitting the flexion data to a single-phase exponential equation. Non-linear regression was used to determine the gliding resistance^32^.

### Tensile Biomechanical Testing

Following gliding function testing, the FDL was released from the tarsal tunnel, and the tibia was removed. The proximal end of the FDL and the digits were held in opposing custom grips in an Instron device (Instron 8841 DynaMight axial servohydraulic testing system, Instron Corporation, Norwood, MA). The tendon was displaced in tension at 30 mm/minute until failure. Stiffness and maximum load at failure were calculated from the force displacement curve.

### Transmission Electron Microscopy

FDL tendons were isolated (n = 3 per diet or genotype) and fixed in Glutaraldehyde Sodium Cacodylate fixative. One-micron axial sections were cut and stained with Toluidine blue. One-micron sections were then trimmed to 70 nm and stained with uranyl acetate and lead citrate. Sections were placed on grids for imaging on a Hitachi 7650 Analytical TEM. Eight to twelve non-overlapping images were taken from mid-substance of each tendon at 15,000x and 40,000x magnification. For measurement of fibril diameter, a region of interest (ROI) of was determined within each image so that a minimum of 80 fibrils could be measured. Diameters were measured along the y-axis. Collagen Fibril density was measured in a 2340 × 1860 pixel area. Collagen fibril density and diameter measurements were made in ImageJ.

### Insulin receptor expression and signaling

Primary tenocytes were isolated from C57Bl/6 J mice and used up to passage 3. Whole FTs were isolated from HFD and LFD-fed mice 48 weeks after diet initiation (n = 3 per group). Tenocytes and tendons were stimulated with either vehicle (0.5% BSA in PBS) or 1 nM insulin for 15 minutes, followed by protein isolation for western blot analyses. Blots were probed for phospho-AKT Ser473 (1:1000, Cell Signaling, #4060), and total AKT (1:1000, Cell Signaling, #9272 S)^33^ and were developed with SuperSignal West Pico or Femto Chemiluminescent Substrate and imaged on a GelDocXR (BioRad, Hercules, CA). To assess changes in IRβ expression in IRcKO^S100a4^ mice, total protein was extracted from WT and IRcKO^S100a4^ tendons (n = 3 per genotype) for western blot analyses. Blots were probed with IRβ (1:300, Santa Cruz, #sc-20739)^34^ and β-actin (1:2500, Sigma Aldrich, #A2228)^35^, and were developed with SuperSignal West Pico or Femto Chemiluminescent Substrate and imaged on a GelDocXR (BioRad, Hercules, CA).

### Statistical Analyses

For obesity/T2DM studies, body weight, blood glucose levels, biomechanical and gliding data were analyzed using a two-way analysis of variance (ANOVA) followed by Bonferroni’s multiple comparisons with significance set at α = 0.05. TEM and body fat percentage were analyzed using one-way ANOVA with Bonferroni’s post-hoc multiple comparisons. Statistical differences between WT and IRcKO^S100a4^ were determined using un-paired t-tests. All data were analyzed using Prism GraphPad 7.0 statistical software. Data are presented as mean ± SEM.

### Data Availability

All data generated or analyzed during this study are included in this article.

## Electronic supplementary material

Supplementary Information
