# Supplementary material for: Obesity/Type II Diabetes Promotes Function-limiting Changes in Murine Tendons that are not reversed by Restoring Normal Metabolic Function
Source: Sci Rep. 2018 Jun 15;8:9218. doi: 10.1038/s41598-018-27634-4 (PMC6003963; doi:10.1038/s41598-018-27634-4)

Supplementary Information

Obesity/ Type II Diabetes Promotes Function-limiting Changes in Murine Tendons that are not reversed by Restoring Normal Metabolic Function

Valentina Studentsova, Keshia M. Mora, Melissa F. Glasner,
Mark R. Buckley, Alayna E. Loiselle

Full unedited gels for Figure 4

Murine Tenocytes

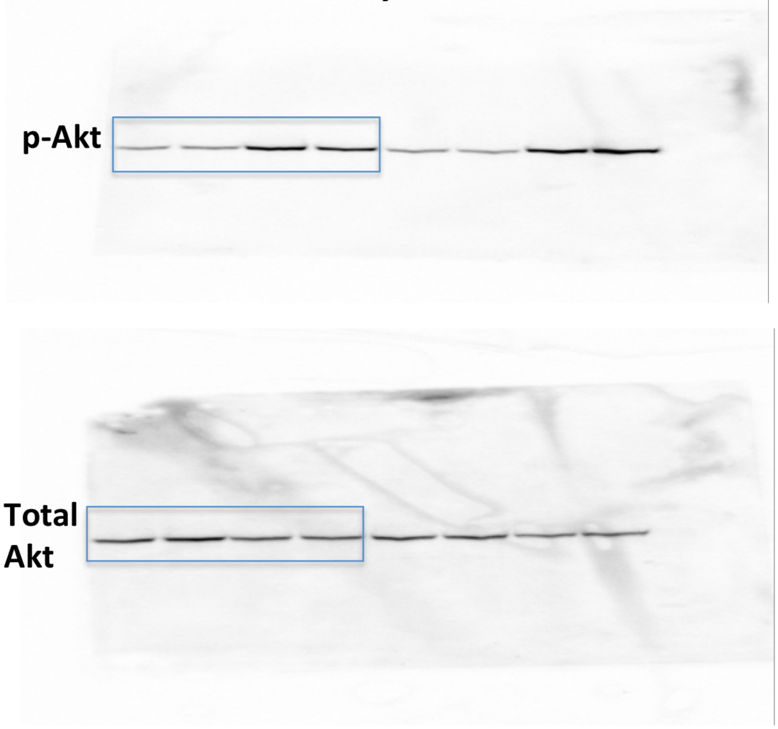

Murine Tendon

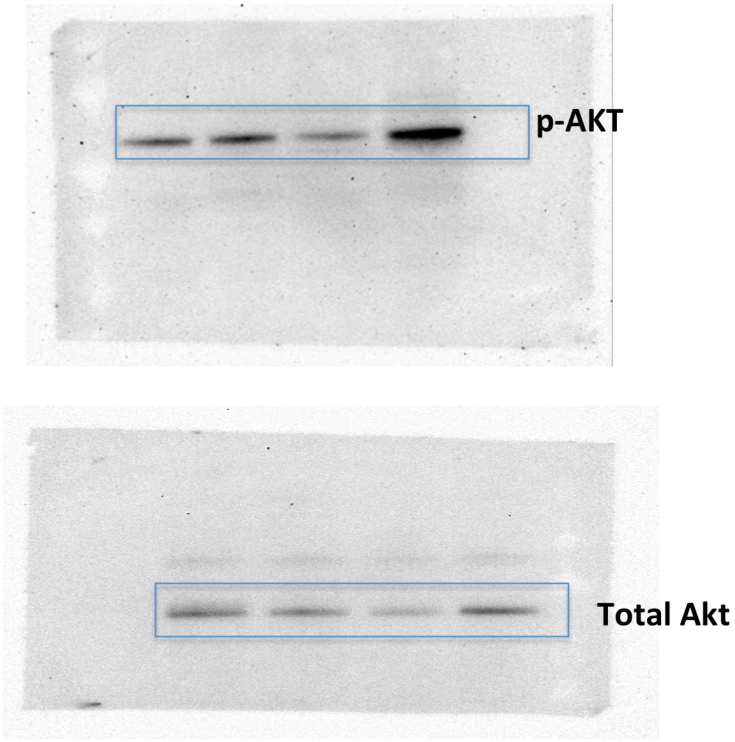

<u>Full unedited gels for Figure 5</u>

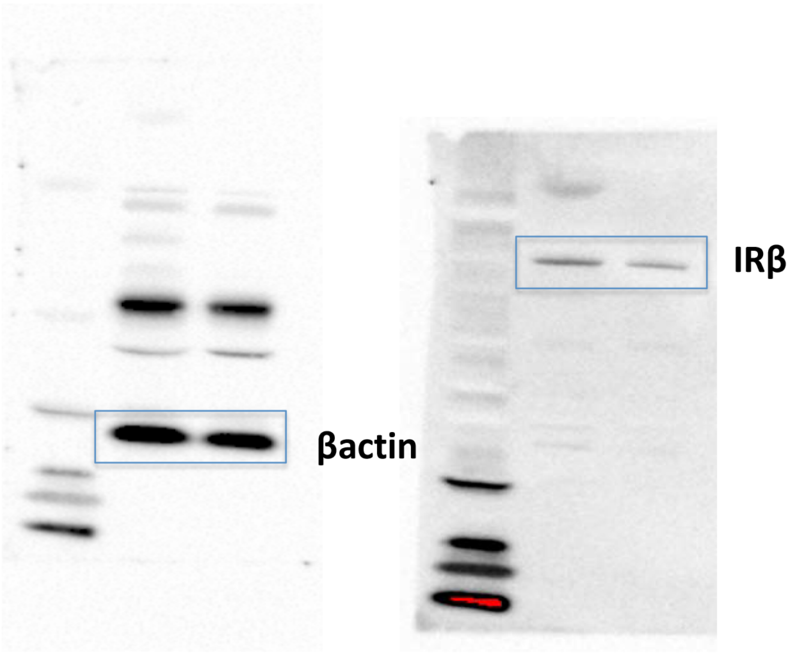

Supplement: Supplementary file 1 — Supplementary Information [file 41598_2018_27634_MOESM1_ESM.pdf]
